# Formulation of the novel structure curcumin derivative–loaded solid lipid nanoparticles: synthesis, optimization, characterization and anti-tumor activity screening *in vitro*

**DOI:** 10.1080/10717544.2022.2092235

**Published:** 2022-07-01

**Authors:** Ke Li, Chao Pi, Jie Wen, Yingmeng He, Jiyuan Yuan, Hongping Shen, Wenmei Zhao, Mingtang Zeng, Xinjie Song, Robert J. Lee, Yumeng Wei, Ling Zhao

**Affiliations:** a Key Laboratory of Medical Electrophysiology, Ministry of Education, School of Pharmacy of Southwest Medical University, Luzhou, China; b Luzhou Key Laboratory of Traditional Chinese Medicine for Chronic Diseases Jointly Built by Sichuan and Chongqing, The Affiliated Traditional Chinese Medicine Hospital of Southwest Medical University, Luzhou, Sichuan, P.R. China; c Central Nervous System Drug Key Laboratory of Sichuan Province, Southwest Medical University, Luzhou, Sichuan, P.R. China; d Clinical Trial Center, The Affiliated Traditional Chinese Medicine Hospital of Southwest Medical University, Luzhou, Sichuan, P.R China; e School of Biological and Chemical Engineering, Zhejiang University of Science and Technology, Hangzhou, Zhejiang, China; f Department of Food Science and Technology, Yeungnam University, Gyeongsan-si, Gyeongsangbuk-do, Republic of Korea; g Division of Pharmaceutics and Pharmacology, College of Pharmacy, The Ohio State University, Columbus, OH, USA

**Keywords:** Curcumin, derivative, solid lipid nanoparticle, anti-tumor activity

## Abstract

This study investigated the effect of structural modification of Curcumin (CU) combined with the solid lipid nanoparticles (SLN) drug delivery system on anti-tumor activity *in vitro*. A new structure of Curcumin derivative (CU1) was successfully synthesized by modifying the phenolic hydroxyl group of CU. CU1 was two times more stable than CU at 45 °C or constant light. The SLN containing CU1 (CU1-SLN) was prepared, and the particle size, polydispersity index, entrapment efficiency, drug loading, and zeta potential of CU1-SLN were (104.1 ± 2.43) nm, 0.22 ± 0.008, (95.1 ± 0.38) %, (4.28 ± 0.02) %, and (28.3 ± 1.60) mV, respectively. X-ray diffraction (XRD) and Differential scanning calorimetry (DSC) showed that CU1 is amorphous in SLN. CU1-SLN released the drug slowly for 48 h, while CU and CU1 were released rapidly within 8 h. In terms of cytotoxicity, CU1 exhibited a 1.5-fold higher inhibition than CU against A549 and SMMC-7721 cells, while CU1-SLN showed 2-fold higher inhibition than CU1. Both CU1 and CU1-SLN reduced the toxicity in normal hepatocytes compared with CU (2.6-fold and 12.9-fold, respectively). CU1-SLN showed a significant apoptotic effect (*p* < 0.05). In summary, CU1 retained the inhibitory effect of CU against tumor cells, while improving stability and safety. Additionally, CU1-SLN presents a promising strategy for the treatment of liver and lung cancer.

## 1. Introduction

Globally, cancer remains the second leading cause of death, about 10 million people died of cancer in 2020 (Cortes et al., 2020; Sung et al., 2021). Currently, chemotherapy remains the primary treatment route for various cancers. However, general chemotherapy is highly toxic and produces side effects, such as bone marrow suppression, neurotoxicity, gastrointestinal reactions, and severe liver damage. Therefore, the discovery and development of a low toxicity and high efficiency drug to treat cancer are of particular importance.

In recent years, active ingredients from natural plants have been extensively studied. Anti-tumor properties have been identified in a large number of monomers derived from natural botanicals (Guo et al., 2020). Curcumin is a plant polyphenol extracted from the rhizome of turmeric (Sahu, 2016). The anti-tumor activity of CU is considered to have great potential in the field of medicine (Shakeri et al., 2019; Lagoa et al., 2020), as it can inhibit the initiation, progression, invasion, and metastasis of tumors through multiple pathways and targets (Kunnumakkara et al., 2008; Feng et al., 2017). However, its clinical application is limited due to the poor selectivity and physicochemical instability of CU (Liu et al., 1553; Peng et al., 2018; Zhao et al., 2019). Nevertheless, because of its precise biological action and relatively simple molecular structure, CU remains an excellent lead molecule for structural modification and anti-tumor drug screening.

The structure-activity relationship shows that CU is composed of two parts: the conjugated β-diketone chain connects two benzene rings and the substituent groups (i.e. hydroxyl and methoxy) on the benzene ring. The phenolic hydroxyl group in the structure is highly polar and prone to losing protons. Literature studies (Talalay & Talalay, 2001) have shown that the phenolic hydroxyl group is crucial in the anti-tumor activity of CU and its derivatives. However, phenolic hydroxyl group is also the main reason for its instability, being oxidized, easily decomposed by light, showing discoloration in an alkaline environment, etc (Rodrigues et al., 2019). CU is prone to biotransformation in the metabolism of the body, and its phenolic hydroxyl group can interact with endogenous substances (glucuronic acid, sulfuric acid, glycine, etc.) and be eliminated (Prasad et al., 2014). Lopes-Rodrigues et al. (2017) obtained a derivative with more stable and stronger anti-tumor activity by modifying the phenolic hydroxyl group of CU. Cao et al. (2014) enhanced the anti-tumor cell proliferation activity of CU through the design of ester bonds. Therefore, proper modification of the phenolic hydroxyl group of CU may increase its stability, and regulate anti-tumor behavior. Nevertheless, the 4-phenolic hydroxyl group of CU is both the active group and the reason for its instability. Therefore, the 4-phenolic hydroxyl group of CU was modified in this research, while the symmetrical phenolic hydroxyl group on the other side was retained. This approach could improve the stability of CU, maintaining its pristine anti-tumor acticity. Thus a new CU derivative, namely CU1 was synthesized. The stability of the derivative CU1 is postulated to be better than that of CU. Simultaneously, since the generated ester bond can be metabolized by esterase to release the drug, the anti-tumor activity can be retained or even enhanced.

In order to further enhance the effects of CU1, a suitable dosage form for new CU derivatives is expected to be identified through modern preparation methods. At present, many reports exist on different dosage forms of CU, such as nanoparticles (Mancarella et al., 2015), liposomes (Moussa et al., 2017), nano-emulsions (Vecchione et al., 2016), microspheres (Tan et al., 2021), and solid dispersions (Mai et al., 2020). Among them, the solid lipid nanoparticle (SLN) is a promising carrier. Since the application of SLN in medicine and gene delivery has been extensively studied, SLN has become a very attractive choice to transport CU1. SLN combines many advantages of nanoparticles and liposomes, and has its own unique advantages (Parhi et al., 2017). In general, SLN has good biocompatibility and is an excellent carrier for drug delivery (Jose et al., 2014; Chetoni et al., 2016). Encapsulating the drug in SLN can effectively improve its pharmacological effects. If specific sustained-release excipients are added, a sustained-release effect can be achieved, to prolong the action time and reduce the number of necessary administrations. In addition, after freeze-drying or spray drying, SLN remains solid at room temperature (RT), so it is not easily affected by the external environment and can improve the stability of drugs that are sensitive to light and humidity, and have unstable chemical structures (Gonçalves et al., 2016; Doktorovova et al., 2017).

Due to the facts mentioned above, CU1 was synthesized and prepared into SLN injection by the membrane-sonic method, to improve the stability, anti-tumor activity and selectivity of CU. The *in vitro* anti-tumor activity of CU1 and CU1-SLN were studied and the results showed that the structural modification and pharmaceutical preparation methods were feasible and promote the clinical application potential of CU.

## 2. Material and methods

### 2.1. Chemical

Curcumin and methyl succinyl chloride were obtained from Chengdu Best Reagent Co., LTD. The Hydrogenated Soybean Phospholipids (HSPC, Lipoid GmbH) and the Polyvinyl Pyrrolidone k15 (PVPk15) were obtained from Luzhou Renkang Biotechnology Co., Ltd.

### 2.2. Cell and cell culture

Human hepatoma cells (SMMC-7721, SK-HEP-1, and HepG2) and human lung cancer cells (A549, A549-T, and H460) were obtained from the Tumor Cell Bank of the Chinese Academy of Medical Sciences, and the normal liver cells (L02) were obtained from the Basic Medicine Laboratory of the Affiliated Hospital of Southwest Medical University. All cell lines were maintained in RPMI 1640 or DMEM (Gibco; Thermo Fisher Scientific, Inc.) with 10% fetal bovine serum (Hyclon, Utah, USA) and 100 U/mL penicillin-streptomycin in a humidified environment with 5% CO_2_ at 37 °C.

### 2.3. Synthesis of CU1

Curcumin (0.1472 g, 0.40 mmol) was added to methylene dichloride (50 mL), and then a solution of triethylamine (96 μL) in methylene dichloride (10 mL) and another solution of methyl succinyl chloride (60 μL, 0.48 mmol) in methylene dichloride (10 mL) were slowly added while stirring, respectively. The reaction mixture was prepared in an ice bath and protected from light. After 30 minutes, the reaction product was purified by silica gel column chromatography to get CU1.

### 2.4. Hydrolytic stability of CU1

CU and CU1 were dissolved in DMSO (dimethyl sulfoxide) at an appropriate concentration, then diluted with PBS (phosphate buffered solution) solution to reach the same concentration, and three aliquots of each were placed in a vial. All samples were placed at 45 °C or under continuous illumination (at 25 °C). The absorbance of CU (425 nm) and CU1 (413 nm) at their respective maximum absorption wavelengths were measured at different time points (0, 0.5, 1, 2, 4, 6, 8, 12, 24, 36, and 48 h), using an ultraviolet spectrophotometer. The ratio of the absorbance at each time point to the absorbance at 0 h was calculated, and the degradation curve of the drug was plotted.

### 2.5. Preparation of CU1-SLN

The solid lipid nanoparticles of CU1 (CU1-SLN) were prepared by thin-film ultrasonic hydration method (Le-Vinh et al., 2021). Specifically, HSPC, PVPk15, and CU1 were dissolved in chloroform and evaporated to dryness in a rotary vacuum evaporator to form a lipid membrane. And then hydrated by ultrapure water and ultrasonically crushed (intensity 50%, 5 min) to obtain uniform nanoparticles. Sucrose was added as a freeze-dried protective agent, the SLN was frozen at −60 °C and lyophilized for 24 h (LGJ-18C; Fourth-Ring Science Instrument plant Beijing Co., Ltd.).

The obtained CU1-SLNs were characterized by tests to measure particle size (PS), polydispersity index (PDI), zeta potential (ZP), encapsulation efficiency (EE), and drug loading (DL). The experimental procedures were repeated at varied ultrasonic disruption intensities, emulsifying temperatures, dosages of excipients, and lipid concentration to drug substance ratios, to optimize the desirable nanoparticle characteristics.

### 2.6. Optimization of CU1-SLN formulation

An orthogonal experimental design was applied οn the basis of a single-factor to screen the optimal formulation of CU1-SLN. The three factors and their three corresponding levels which influence the formulation of CU1-SLN were selected according to the L9 (3 (3)) orthogonal table (Table 1), including the amount of CU1, HSPC and PVPk15. According to the comprehensive score (X), intuitive analysis and variance analysis, the best formula was obtained.

$$\text{Comprehensive score }(X)=X_{n}/X_{\max}\times 90+Y_{n}/Y_{\max}\times 10$$

where *X_n_* and *Y_n_* were EE% and DL% under the *n*-th formulation; the *X_max_* and *Y_max_* were the maximum values of EE% and DL%.

**Table 1.** The factors and levels of orthogonal design.

Levels	Foctors		
	A (CU1, mg)	B (HSPC, mg)	C (PVPk15, mg)
1	7.0	35.0	20.0
2	6.0	30.0	15.0
3	5.0	25.0	10.0

### 2.7. Physicochemical characterization

#### 2.7.1. Particle properties

The PS, PDI, and ZP of CU1-SLN were measured using Malvern Zetasizer-Nanoseries-ZS90 (Malvern, UK). The CU1-SLN suspension was appropriately diluted for the measurement.

#### 2.7.2. EE% and DL%

In an aqueous medium, hydrophobic CU1 precipitates when centrifuged at low speed, whereas CU1 encapsulated in SLNs does not (Feng et al., 2020). EE% was determined by measuring CU1 content in the same volume of CU1-SLN suspension before and after low speed centrifugation (4000 rpm, 10 min). The supernatant CU1-SLN dispersion was demulsified with a mixed solution of methanol and ethyl acetate (4:1, v/v), and tested for the entrapped drug at 413 nm by UV-spectrophotometer (Aoyi Instruments, China) (Bayat et al., 2021). The total drug amount in SLN suspension (0.1 mL) was also determined by the UV-visible spectrophotometer, as described above, following dissolution in a mixed solution of methanol and ethyl acetate. The lyophilized liposome powder was accurately weighed, dissolved in a mixed solution of methanol and ethyl acetate, demulsified by ultrasound, and the proportion of the drug was determined (DL%).

$$\text{EE \% }=\;W_{e}/W_{t}\times 100$$

$$\text{DL \% }=\;W_{t}/W_{0}\times 100$$

where *W_t_* was the total content of the CU1; *W_e_* was the content of the CU1 encapsulated in SLN; and *W_0_* was the total weight of SLN, including drug and excipients.

#### 2.7.3. Transmission electron microscopy (TEM)

A drop of CU1-SLN dispersion was placed over a 200-mesh copper grid, stained with phosphotungstic acid (2%, w/v) and dried at RT before TEM imaging (H-7500; Hitachi Ltd., Tokyo, Japan).

#### 2.7.4 X-Ray powder diffraction (XRD)

XRD analyses of CU1, blank SLN, and CU1-SLN were performed by XD/MAX2500/PC diffractometer (Rigaku Corporation, Japan). Each sample was scanned from 5° to 90° in 2θ with a step size of 0.026°.

#### 2.7.5. Differential scanning calorimetry (DSC)

DSC scan was performed by a microcalorimeter (NETZSCH TG-DSC STA-449 F3, Germany). Samples were heated in the temperature range of 30 − 400 °C on an aluminum pan at a rate of 10 °C/min, under nitrogen atmosphere.

#### 2.7.6. Drug Release properties

The *in vitro* release of CU1 from the SLN was measured by a dialysis membrane method (Le-Vinh et al., 2021). Briefly, CU1-SLN suspension, including 400 μg of CU1, was dispersed in physiological saline solution and transferred to the dialysis bag (MWCO 8,000–14,000). The dialysis bag was immersed in the physiological saline solution medium (200 mL, containing 5% Tween 80) at 37 ± 0.5 °C, under paddle rotation of 100 rpm/min. Samples (4 mL) were withdrawn from the release medium at predetermined time points and replaced by the same volume of fresh medium with the same temperature. Quantification of the CU1 released from the SLN was performed using the UV-visible spectrophotometer.

*In vitro* drug release data were fitted to various kinetic models and regression coefficient value (r (2)) was calculated.

$$\mathrm{Zero}-\mathrm{order}:\;M_{t}=\;k_{0}t\;+\;b$$

$$\mathrm{First}-\mathrm{order}:\;\ln(100-\;M_{t})\;=\;-k_{1}t\;+\;b$$

$$\mathrm{Higuchi}:\text{ Mt }=\;k_{h}{\text{t }}^{1/2}+\;b$$

where *M_t_* was the cumulative release at time *t*; the *k_0_, k_1_*, and *k_h_* were the rate constants.

### 2.8. In vitro cytotoxicity assay

The MTT ([3-(4,5-dimethylthiazole-2-yl)-2,5-diphenyltetrazolium bromide]) method was employed to determine the effects of the SLN preparation on hepatoma cells (SMMC-7721, SK-HEP-1, HepG2), lung cancer cells (A549, A549-T, H460) and normal liver cells (L02). Cells were seeded in 96-well plates at a density of 5000 cells per well and incubated for 24 h. The cells were then treated with media containing different drugs (at concentrations of 6.25, 12.5, 25, 50, and 100 μg/mL). After 24 h, 48 h, 72 h, and 96 h incubation, respectively, 20 μL MTT (0.5 mg/mL) were added to each well, and the plates were incubated for another 3 h. The absorbance of the MTT derivative dissolved in 150 μL of dimethyl sulfoxide was measured by a microplate reader (Spectra Max M3, Molecular Devices, USA) at 490 nm. The entire experiment was carried out in triplicate. The concentration of the drug causing 50% growth inhibition (IC50) was calculated.

$$\mathrm{Cell}\;\mathrm{inhibition}=\;[1-(\frac{A_{\mathrm{sample}}-A_{\mathrm{blank}}}{A_{\mathrm{control}}-A_{\mathrm{blank}}})]\times 100$$

### 2.9. Cell Uptake study

The laser scanning confocal microscope (LSCM) was used to assess the intracellular uptake efficiency of CU1. The following protocol was performed for cellular internalization. Cells were seeded in acid-etched coverslips kept in 24-well plates with a density of 24000 cells/coverslip. The culture medium was discarded after 24 h and treated with drug-containing medium for another 24 h. Then, the cells were washed twice with PBS to remove non-intracellular drugs and 4% paraformaldehyde (400 μL) was added to fix the cells. Finally, the cells were stained with DAPI and imaged under the LSCM (Leica TCS SP8).

### 2.10. Cell Apoptosis assay

Cell apoptosis was evaluated using the Annexin V-FITC apoptosis detection kit (BD Biosciences, USA) according to the instructions of the manufacturer. Briefly, cells were seeded into 6-well plates, incubated overnight, and then treated with the test drug (10, 15, and 20 μg/mL) for 48 h. Cells from each sample were suspended in 500 μL of Annexin V binding buffer. Annexin V-FITC (5 μL) and 5 μL of propidium iodide (PI) were added and incubated for 15 min in the dark. The stained cells were analyzed by flow cytometry using a FACS Calibur (BD Biosciences, San Jose, CA, USA). Each test was performed in triplicate.

## 3. Results

### 3.1. Synthesis of CU1

**Figure 1.**
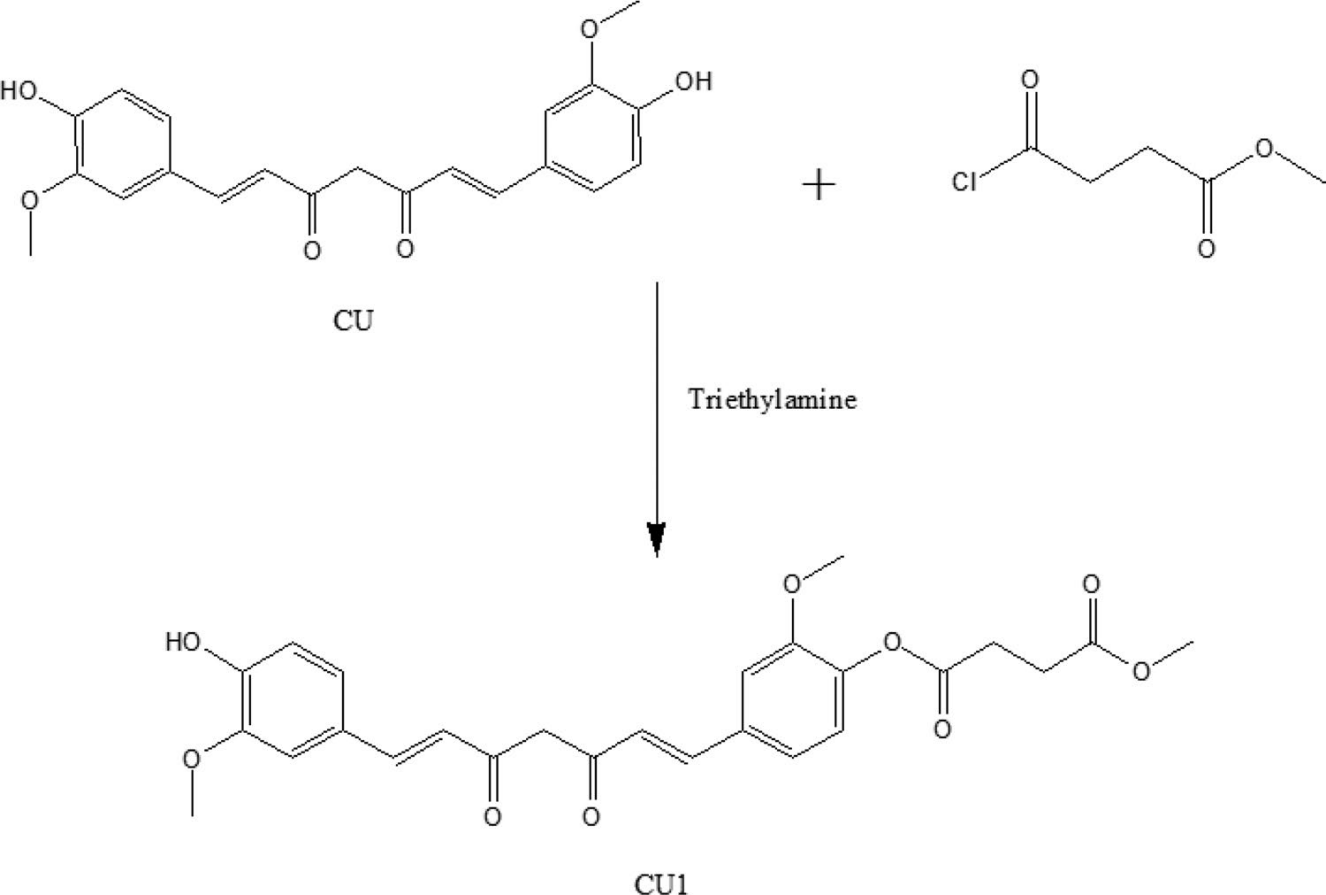
The synthetic route of CU1.

The synthesis route of CU1 is illustrated in Figure 1. CU1 was characterized as follows: 2-hydroxy-5-((1E,6E)-7-(4-hydroxy-3-methoxyphenyl)-3,5-dioxohepta-1,6-dien-1-yl) phenyl methyl succinate: dark-yellow powder, 95% yield. 1H NMR (500 MHz, DMSO): 16.25 (s, 1H), 9.70 (s, 1H), 7.61 (d, J = 3.5 Hz, 1H), 7.58 (d, J = 4.0 Hz, 1H), 7.51 (s, 1H), 7.34 (s, 1H), 7.31 (d, J = 8.5 Hz, 1H), 7.17 (d, J = 8.0 Hz, 1H), 7.12 (d, J = 8.5 Hz, 1H), 6.95 (d, J = 16.0 Hz, 1H), 6.84 (s, 1H), 6.81 (d, J = 4.5 Hz, 1H), 6.78 (s, 1H), 3.84 (s, 6H), 3.63 (s, 3H), 2.84 (t, J = 13 Hz, 2H), 2.66 (t, J = 13.5 Hz, 2H). Electrospray ionization mass spectrometry (ESI-MS) m/z: 483.16 [M + H]+.

### 3.2. Hydrolytic stability of CU1

**Figure 2.**
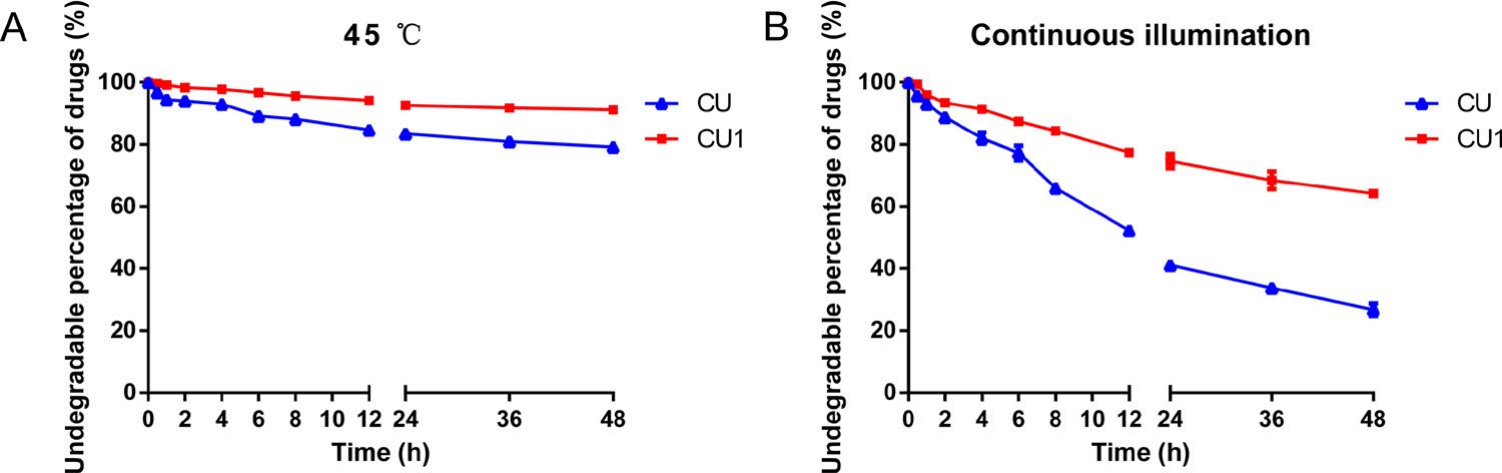
(A) The respective degradation curves of CU and CU1 solutions at 45 °C. (B) The degradation curves of CU and CU1 solutions under continuous light, respectively. (means ± SD, *n* = 3).

Storage in a higher temperature environment significantly degraded the drugs in the samples. After 48 h, the drug content of the CU solution was reduced by about 21%, while CU1 was only reduced by 9% (Figure 2(a)). CU was extremely unstable when exposed to light, so after 48 hours of continuous light, only about 26% of the drug remained in the CU solution. The drug content in the CU1 solution was as high as 64%, which was about 2.5 times higher than the content of CU (Figure 2(b)), showing that CU1 greatly improved the stability of CU.

### 3.3. Optimization of CU1-SLN

Table 2 shows the orthogonal experimental results. Intuitive analysis indicated that according to the R-value, the effects of each factor on the comprehensive score (X) were in the following order: (B)HSPC > (A)CU1 > (C)PVPk15. K1/3, K2/3, and K3/3 represented the average of each level. The results of the three-factor analysis showed that the optimal parameters of CU1-SLN were A2B1C2. As shown in Table 3, the analysis of variance results showed that factor A and factor B had significant effects on EE% and DL% of CU1-SLN (*p* < 0.05), which was consistent with the results of the intuitive analysis.

**Table 2.** Results of orthogonal design test.

No.	A	B	C	EE (%)	DL (%)	X
1	1	1	1	80.9	4.41	88.2
2	1	2	2	79.7	4.81	87.9
3	1	3	3	71.4	3.27	76.6
4	2	1	2	90.2	4.59	97.7
5	2	2	3	88.7	5.04	97.2
6	2	3	1	81.4	3.82	87.6
7	3	1	3	91.6	3.79	97.5
8	3	2	1	87.1	3.58	92.7
9	3	3	2	83.6	2.99	88.1
K1/3	252.7	283.5	268.5			
K2/3	282.4	277.7	273.7			
K3/3	278.3	252.3	271.3			
R	29.7	31.2	5.2			

X, comprehensive score; R, the range analysis; K1/3, K2/3, and K3/3 represented the average of each level.

**Table 3.** Results of variance analysis.

No.	Sum of squares	Free degree	Mean square	F value	P value
A	173.499	2	86.724	19.886	< 0.05
B	183.202	2	91.601	21.004	< 0.05
C	4.516	2	2.258	0.518	> 0.05
Errors	8.722	2	20.408		

**Figure 3.**
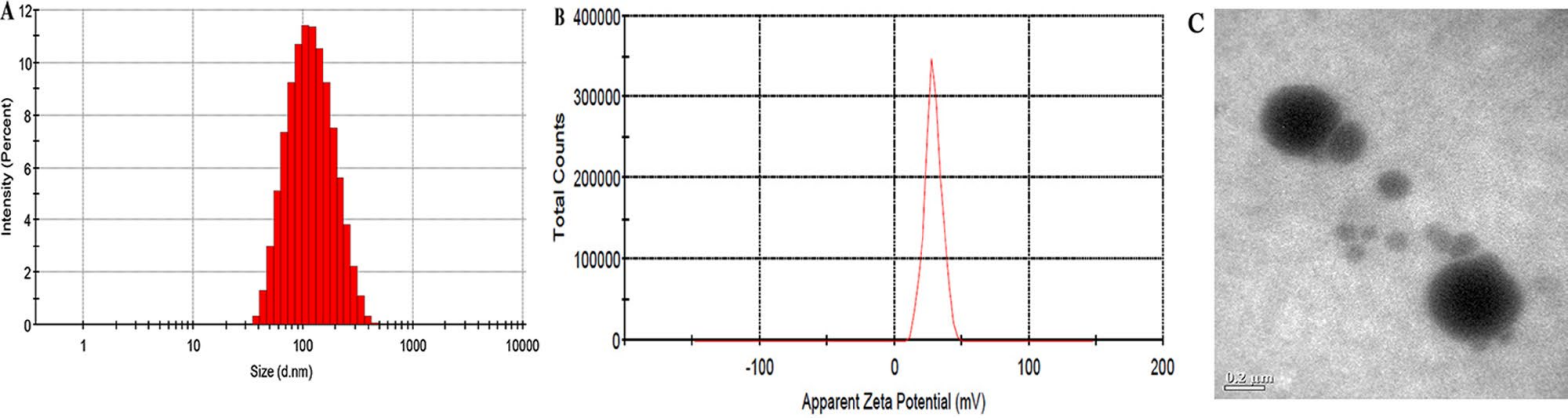
Verification test results of samples in size (A), zeta potential (B), and TEM image (C) of CU1-SLN.

Three batches of samples were prepared using the optimal formulation for the assessment of the following characteristics. Specifically, the amounts of CU1, HSPC, and PVPk15 in the optimized SLN prescription were 6 mg, 35 mg, and 15 mg, respectively. The optimal content of the freeze-dried protectant (sucrose) was 1.25% (w/v). Three batches of CU1-SLN samples were prepared using the optimal method (Figure 3 and Table 4). The average PS and ZP were 104.1 ± 2.43 nm and 28.3 ± 1.60 Mv, respectively. The PDIs of three batch samples were 0.229 ± 0.004, 0.217 ± 0.003 and 0.213 ± 0.005, respectively. All of these were below 0.3, meeting the standard. This indicates that the optimized prescription had uniform particle size distribution. The average EE and DL were 95.1 ± 0.38% and 4.28 ± 0.02%, respectively. CU1-SLN was spheroidal in shape and uniform in size, and there was basically no adhesion between the particles (Figure 3(c)). The results indicated that the preparation method of CU1-SLN had good reproducibility.

**Table 4.** Verification test results of three batch of samples (means ± SD, *n* = 3).

No.	Particle size (nm)	PDI	Zeta (mV)	EE (%)	DL (%)
1	101.4 ± 0.21	0.229 ± 0.004	26.8	95.1	4.25
2	106.1 ± 0.45	0.217 ± 0.003	28.2	95.7	4.30
3	104.8 ± 0.70	0.213 ± 0.005	30.0	95.8	4.28
$\bar{x}$ ± s	104.1 ± 2.43	0.220 ± 0.008	28.3 ± 1.60	95.5 ± 0.38	4.28 ± 0.02

### 3.4. XRD and DSC

**Figure 4.**
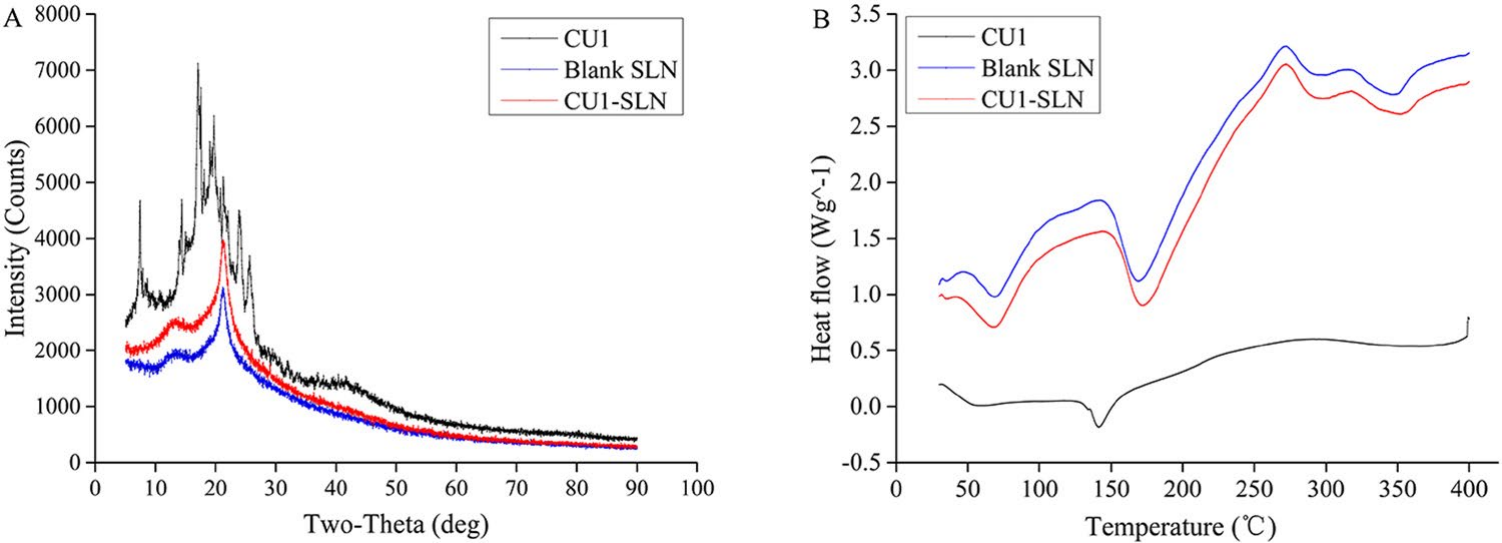
XRD pattern (A) and DSC pattern (B).

Pure drug, blank and drug-loaded SLN were analyzed with XRD. Figure 4(a) shows the XRD of CU1, blank SLN, and CU1-SLN. The crystalline CU1 had a series of diffraction peaks, while they disappeared in the diffraction patterns of CU1-SLN. This indicates that CU1 might not exist in crystal form in SLN.

DSC analysis was used to investigate the crystal structure of CU1 in SLN (Figure 4(b)). CU1 has an obvious endothermic peak at about 145 °C, indicating the crystalline structure of the drug. This peak was completely absent in the curve of CU1-SLN, where two exclusively endothermic peaks appeared corresponding to the endothermic curve of empty SLN (HSPC: 50–100 °C, PVPk15: 150–200 °C). This thermal behavior suggested that CU1 was completely suspended inside the matrix of the SLN and it was present in the amorphous form in SLN, which corroborated the results of XRD.

### 3.5. In vitro drug release study

**Figure 5.**
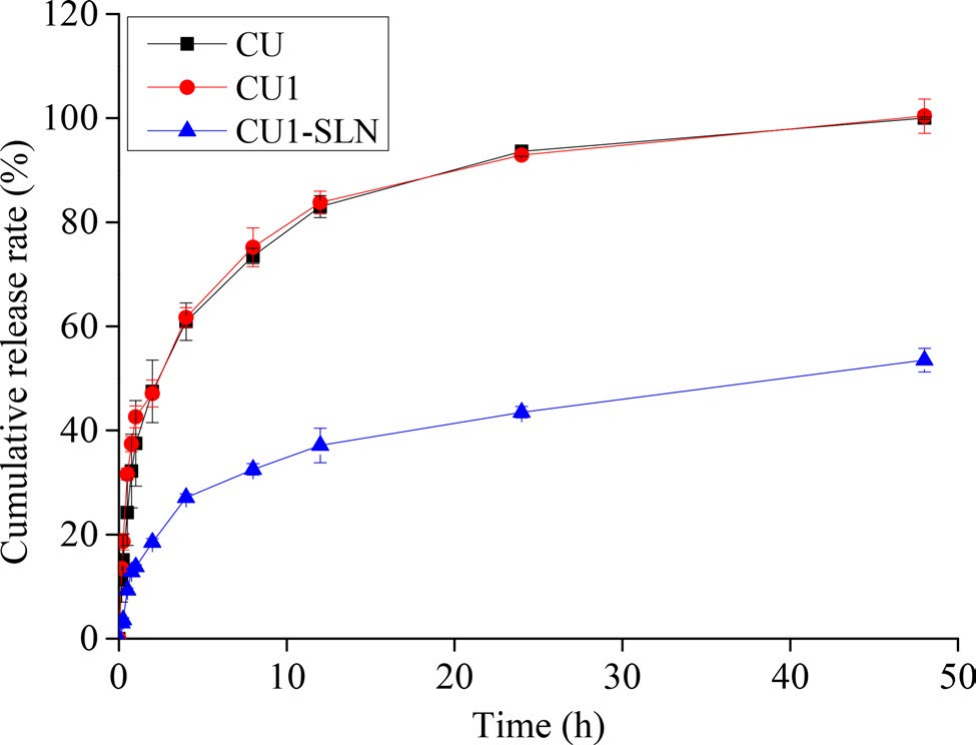
Cumulative release rate (%) *in vitro* of CU, CU1, and CU1-SLN for 48 h (means ± SD, *n* = 3).

The drug release of CU, CU1, and CU1-SLN is shown in Figure  5. In the same release medium, the cumulative release amount of CU and CU1 was similar, and the release was faster in the first 8 h and almost completed at 48 h. In the case of CU1-SLN suspension, a fast drug release was observed in the first 8 h, and only 53.5 ± 2.3% of CU1 in CU1-SLN was released in the phosphate buffer at the end of 48 h. The fitting results of the drug release kinetic model showed that the release of the three groups of drugs all fit the first-order kinetic equation (Table 5). Nevertheless, the Higuchi model also showed relatively good correlation in the case of CU1-SLN. This result shows that CU1-SLN has a good sustained-release effect.

**Table 5.** Drug release kinetic parameters and fitting coefficients.

	Zero order equation		First-order equation		Higuchi’s equation	
	r (2)	k_0_	r (2)	k_1_	r (2)	k_h_
CU	0.58375	1.85082	0.9389	0.43574	0.85135	14.9196
CU1	0.57119	1.77306	0.90613	0.11487	0.83831	14.32669
CU1-SLN	0.71125	1.03972	0.93155	0.2336	0.92831	7.99967

r^2^: fitting coefficient; k_0_, k_1_, and k_h_ are the rate constant.

### 3.6. Cytotoxicity assay

**Figure 6.**
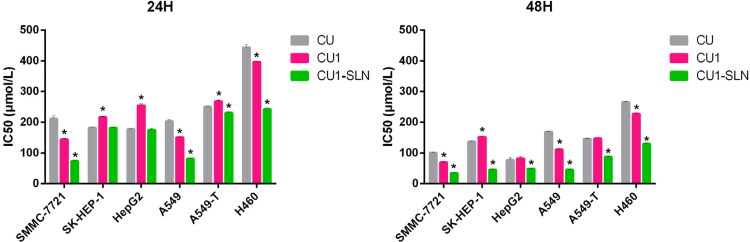
IC50 values of three drugs against cancer cells proliferation at 24 h (A), 48 h (B), 72 h (C), and 96 h (D), respectively (means ± SD, *n* = 3). *p < 0.05, there was a significant difference compared with CU.

**Figure 7.**
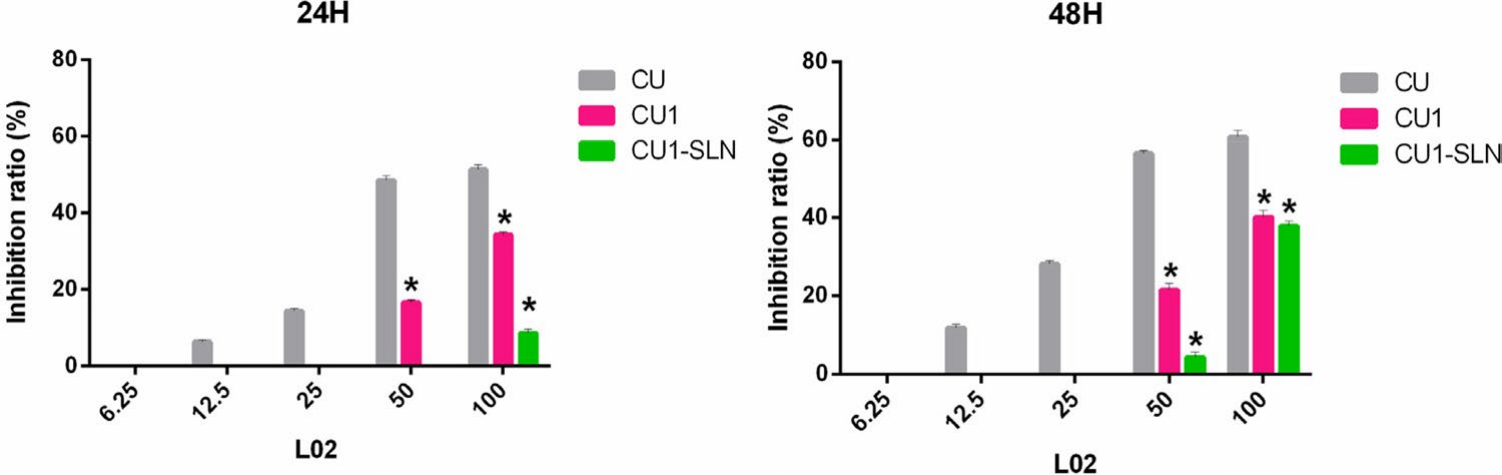
The inhibition ratio of three drugs against the normal hepatocyte L02 proliferation at 24 h and 48 h, respectively (means ± SD, *n* = 3). *p < 0.05, there was a significant difference compared with CU. Note: At some concentrations there was no inhibitory effect, so they were not shown in the figure.

The MTT method was employed to determine the toxic effect of CU, CU1, and CU1-SLN on human hepatoma cells (SMMC-7721, SK-HEP-1, and HepG2), human lung cancer cells (A549, A549-T, and H460), and normal liver cells (L02). The IC50 results of CU, CU1 and CU1-SLN at two different time points (24 h, 48 h) are shown in Figure 6 and Figure 7. For tumor cells, lower IC50 values are associated with better drug effect. CU, CU1, and CU1-SLN can all inhibit the proliferation of the human hepatoma cells and lung cancer cells studied in this experiment, and this inhibitory effect is concentration- and time- dependent (Figure 6).

#### 3.6.1. Toxic effects on human hepatoma cells

The IC50 values of the three drugs gradually decreased over time for three different hepatoma cells, indicating that the inhibitory effect increased as the action time prolonged, showing the time dependence of the drugs. Additionally, higher drug concentrations were associated with higher inhibition rates, suggesting that the effect of the drug is concentration-dependent.

The IC50 value of CU in SMMC-7721 cells was approximately 1.5-fold higher than that of CU1 (IC50: 210.96 μM vs. 144.12 μM at 24 h, 100.43 μM vs. 69.43 μM at 48 h). This indicates that the inhibitory effect of CU1 on hepatoma SMMC-7721 cells was significantly enhanced in comparison to CU. However, after CU1 was loaded in SLN, this inhibition was further enhanced, the IC50 value of CU1 was about twice higher than that of CU1-SLN (IC50: 144.12 μM vs. 73.51 μM at 24 h, 69.43 μM vs. 34.51 μM at 48 h).

The cytotoxicity analysis of CU, CU1, and CU1-SLN on the other two hepatoma cells, HepG2 cells and SK-HEP-1 cells showed that CU1 was less potent than CU in terms of IC50 values at 24 h. However, the gap between CU1 and CU narrowed at 48 h. After CU was modified into CU1, the sensitivity to HepG2 cells and SK-HEP-1 decreased, but it still retains a certain inhibitory effect. Nonetheless, CU1-SLN still significantly enhanced the inhibitory effect of CU1 on cancer cells and exhibited better anti-tumor cell proliferation effects than CU. After 48 h of treatment, the inhibitory effect of CU1-SLN on HepG2 was increased 1.6-fold (IC50: 76.27 μM vs. 48.48 μM at 48 h), and the inhibitory effect on SK-HEP-1 was increased 3-fold compared with CU (IC50: 136.96 μM vs. 45.018 μM at 48 h).

#### 3.6.2. Toxic effects on human lung cancer cells

The IC50 values of the three drugs showed concentration- and time-dependent behavior for three different lung cancer cell lines.

It can be seen from Figure 6 that after CU was modified to CU1, its inhibitory effect on lung cancer A549 cells was significantly improved, and the IC50 value of CU1 was lower than the IC50 value of CU at all time points. Furthermore, CU1-SLN enhanced this inhibitory effect. Compared with CU1, the inhibitory effect of CU1-SLN on the proliferation of tumor cells was about 2-fold higher (IC50: 150.90 μM vs. 81.12 μM at 24 h, 111.13 μM vs. 45.23 μM at 48 h). This result showed that CU1 was more sensitive to lung cancer A549 cells. Additionally, after CU1 was made into CU1-SLN, its inhibition rate could be increased again, suggesting that CU1 and CU1-SLN may also have a certain selective inhibitory effect.

The analysis on the inhibitory effect of the other two lung cancer cell lines indicated that the inhibitory effect of CU1 on the lung adenocarcinoma paclitaxel-resistant cell line A549-T resembled the effect of CU, while a slight advantage was observed over the H460 cell line. Modification of CU to CU1 retained the good inhibitory effect of CU on these two types of cancer cells. Although the inhibitory effect of CU1 on cancer cells did not significantly improve, the effect was significantly improved after CU1 was made into CU1-SLN. At 48 h, the IC50 value of CU1-SLN on A549-T was reduced 1.7-fold compared with CU1 (IC50: 147.16 μM vs. 87.28 μM at 48 h), and the IC50 value on H460 was reduced 2-fold (IC50: 227.46 μM vs. 129.25 μM at 48 h).

#### 3.6.3. Toxic effects on normal liver cells

The IC50 value could not be calculated within 48 h, due to the low toxicity of CU1 and CU1-SLN to L02 cells, so the inhibition ratio (%) was chosen to display the results (Figure 7). Figure 7 shows that the order of toxicity to L02 within 48 h is CU < CU1 < CU1-SLN, and especially within 24 h, the effect of CU1-SLN is negligible. Taking the concentration of 50 μg/ml as an example, at 24 h, the toxicity of the CU group was 2.9-fold higher than that of the CU1 group (inhibition rate: 48.47% vs 16.50% at 24 h), while no obvious toxicity was found in the CU1-SLN group at the same time. At 48 h, the toxicity of CU continued to increase, which was 2.6-fold higher than that of CU1 (inhibition rate: 56.53% vs 21.57% at 24 h), and 12.9-fold higher than that of CU1-SLN (inhibition rate: 56.53% vs 4.37% at 24 h). Overall, CU1 was much less toxic to normal hepatocytes than CU, which means that after CU was modified to CU1, the safety was significantly improved (*p* < 0.05), and this advantage became more pronounced over time. In addition, CU1-SLN did not increase the toxicity of the drug and showed a better safety profile than CU1.

### 3.7. Cell uptake study

**Figure 8.**
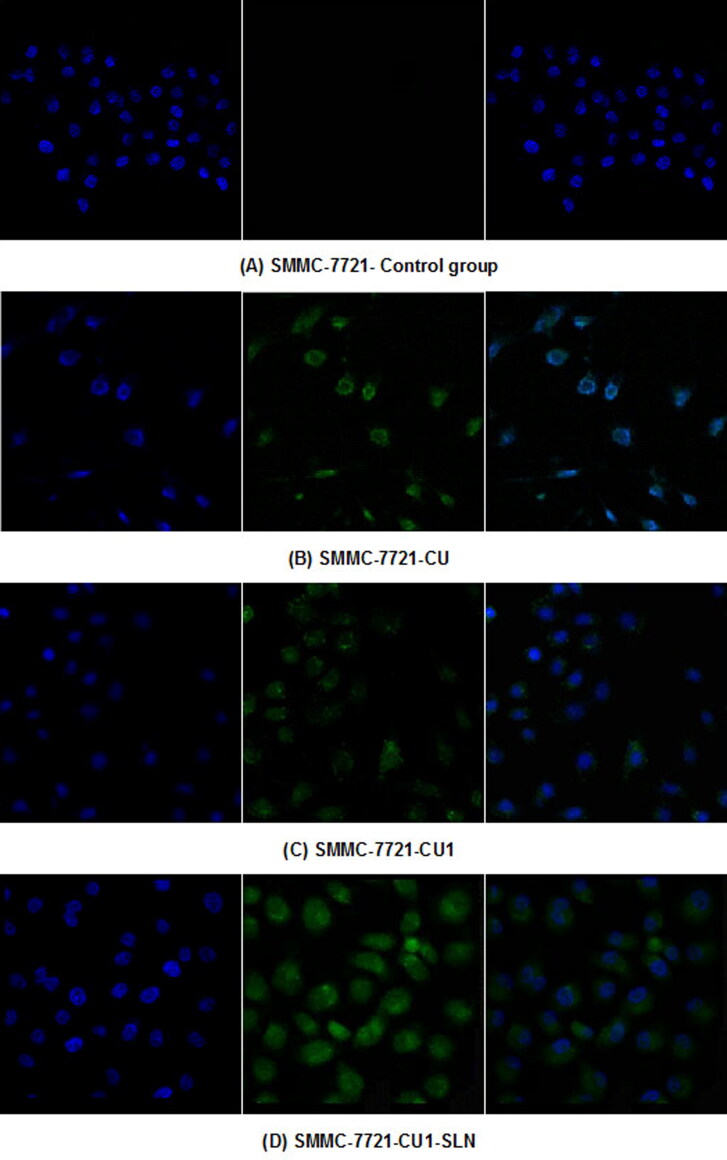
Drug distribution in SMMC-7721 cells. (A) Control group, (B) CU, (C) CU1, and (D) CU1-SLN.

**Figure 9.**
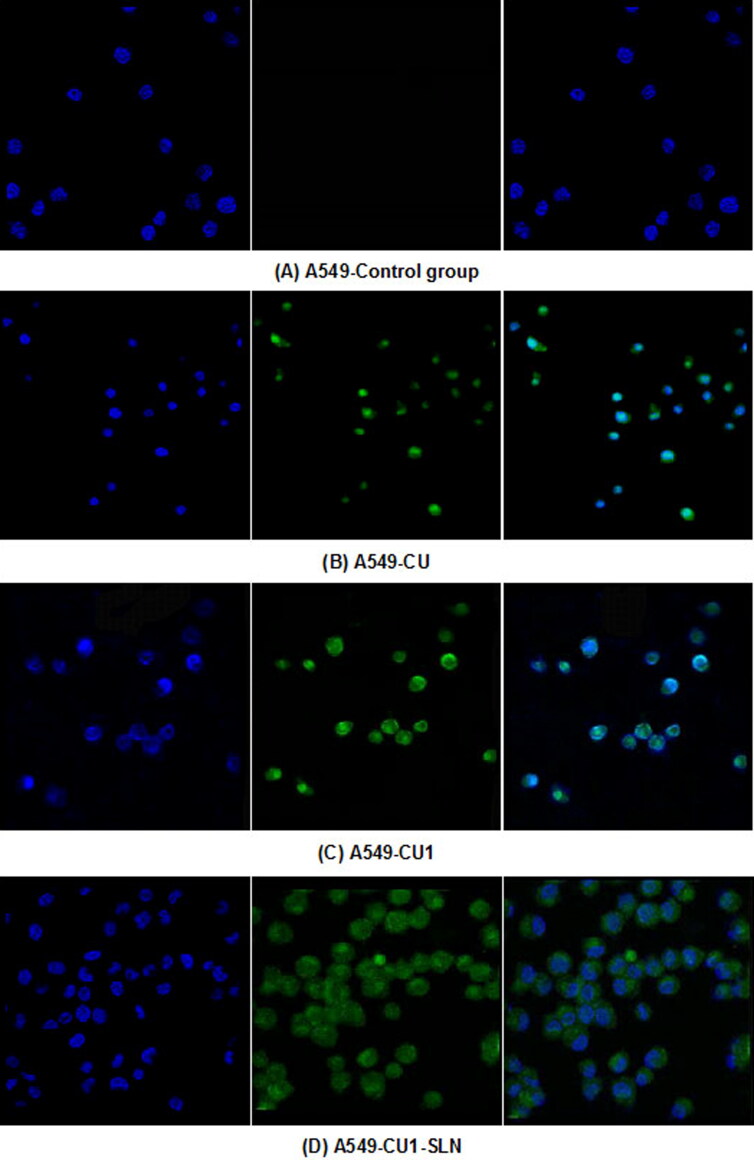
Drug distribution in A549 cells. (A) Control group, (B) CU, (C) CU1, and (D) CU1-SLN.

The distribution of the drug in hepatoma SMMC-7721 cells and lung cancer A549 cells was detected, through LSCM (Figure 8 and Figure 9). From left to right, the fluorescence images of each group show the blue fluorescence of the DAPI stained cell nucleus, the green fluorescence of the drug, and the combined image, respectively. Hepatoma SMMC-7721 cells and lung cancer A549 cells were administered to CU, CU1 and CU1-SLN, respectively. After 24 h of action, some amount of drugs could be detected in the nucleus, indicating that the drug mainly entered the nucleus of cancer cells to exert anti-tumor effects. The drug was distributed in the entire cell nucleus, while a small amount was also present in the cytoplasm. The experimental results suggested that liposomes had a good affinity with cells and could effectively carry drugs into cells.

### 3.8. Apoptosis

**Figure 10.**
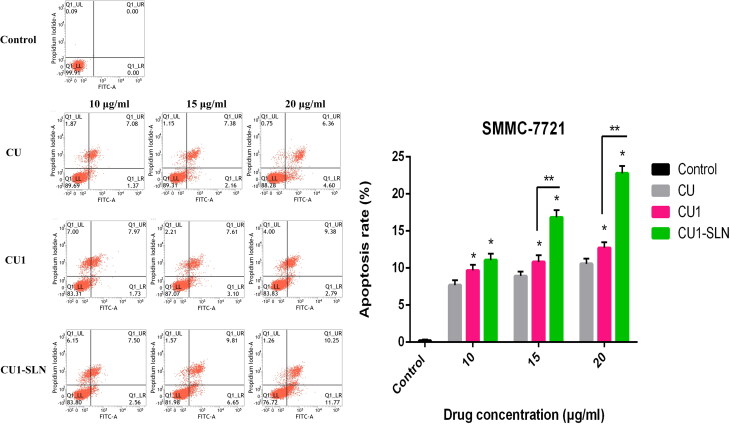
Apoptosis of CU, CU1, and CU1-SLN against SMMC-7721 cell at 48 h, and the statistical analysis of the total apoptosis rate of SMMC-7721 cells (means ± SD, *n* = 3). * vs CU, *p* < 0.05; ** vs CU1, *p* < 0.05.

**Figure 11.**
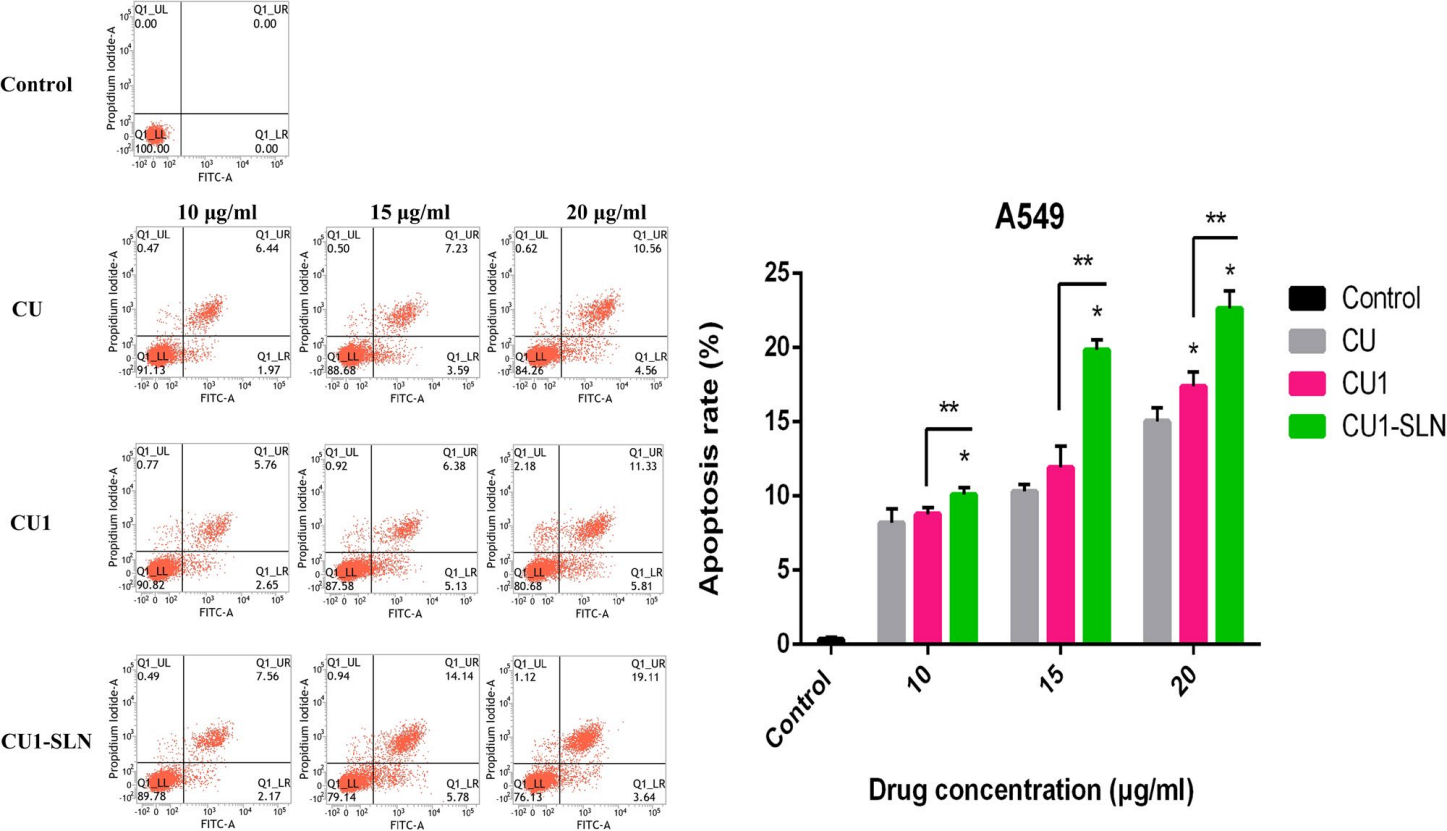
Apoptosis of CU, CU1, and CU1-SLN against A549 cell at 48 h, and the statistical analysis of the total apoptosis rate of A549 cells (means ± SD, *n* = 3). * vs CU, *p* < 0.05; ** vs CU1, *p* < 0.05.

Figure 10 shows that CU, CU1, and CU1-SLN can induce the apoptosis of hepatoma SMMC-7721 cells in a concentration-dependent manner. Higher concentration of the drug induced the stronger apoptotic effect of the cells. Under the same concentration of drugs, the apoptotic effect strength was as follows: CU < CU1 < CU1-SLN. Compared with CU, CU1-SLN mainly induced early apoptosis of hepatoma SMMC-7721 cells. As shown in Figure 11, all three drugs induced apoptosis of lung cancer A549 cells, and the apoptotic effect was also concentration-dependent. The apoptotic effect strength of the same concentration of drug was: CU < CU1 < CU1-SLN. CU1-SLN mainly induced late-stage apoptosis of lung cancer A549 cells, compared with CU.

## 4. Discussion

As a polyphenol compound, CU has a broad-spectrum anti-tumor effect which can effectively inhibit the proliferation of tumor cells and prevent the invasion and metastasis of malignant tumor cells (Perrone et al., 2015; Panda et al., 2017). However, the low stability of CU considerably hinders the development and application of CU. The phenolic hydroxyl group, as the active group of CU, is also one of the reasons for the instability of CU. According to literature studies, chemically modifying the phenolic hydroxyl group in the structure can boost its anti-tumor effectiveness by changing its stability and increasing cell membrane penetration (Rodrigues et al., 2019). Therefore, to achieve the preservation of efficacy and the improvement of stability, the phenolic hydroxyl group of CU was modified by forming ester derivatives in this study, while the phenolic hydroxyl group in the symmetrical position on the other side was retained. Dichlorosulfoxide is a commonly used reagent for esterification with phenolic hydroxyl groups, but it has great toxicity (Zhang et al., 2011). Considering this problem, the methyl succinyl chloride, an organic solvent with low toxicity and easy removal, was selected as an acylation reagent to react with CU. In addition, pre-experiments have found that mixing CU and acylating reagents directly did not cause a reaction without a catalyst. And two different catalysts, namely 4-dimethylaminopyridine (DMAP) and triethylamine were tested (data not shown). The catalytic efficiency of DMAP was lower than that of triethylamine, which may be due to the fact that triethylamine is more basic than DMAP (Majhi et al., 2010; Manju and Sreenivasan, 2012). In an alkaline environment, acylating reagents can easily react with phenolic hydroxyl groups to form esters, and triethylamine could be easily removed by blowing dry nitrogen because of its volatility.

In this study, CU1 was successfully synthesized for the first time with a novel structure, which has not been reported in other literature. The results of hydrolytic stability experiments showed that CU1 can significantly improve the stability of CU, under higher temperature or light conditions. This result confirms the advantage of modifying the phenolic hydroxyl group. Furthermore, SLN has good biocompatibility and can also improve the stability and pharmacological activity of the drug. (Ikeuchi-Takahashi et al., 2016; Khan et al., 2019; Sabapati et al., 2019) Therefore, the CU1-loaded SLN was prepared. A large number of single-factor experiments in the early stage were performed to preliminarily screen the dosage of drugs and excipients in the formula. As the main film material and a common phospholipid for the preparation of SLN, the increase of HSPC content did not affect PS and PDI, but promoted the encapsulation of CU1. In addition, as a surfactant, PVPk15 is helpful to inhibit the crystallization of drugs and adjust the solubility of insoluble drugs in the preparation (Zhang et al., XXXX). When the content of PVPk15 was reduced to 5 mg, some precipitation of CU1 was observed, and the EE% of CU1-SLN was low. Therefore, a optimal amount of PVPk15 was necessary. The results of single-factor experiments showed that EE% of CU1-SLN was a parameter greatly influenced by excipients. When the ratio of excipient to drug was increased, although the EE% improved, the lower DL% would affect the administered dose and there might be redundant empty SLNs. For the nanoscale drug delivery system of SLN, EE% and DL% are highly essential indicators in the characterization, and a good SLN can encapsulate as many active drugs as possible into the carrier. Therefore, EE% was selected as the primary evaluation index of an orthogonal experiment. And the DL% should also be considered.

After optimization, the PS of CU1-SLN prepared in this study was small, uniform, and within the diameter range of most clinically approved nanoparticles (i.e. from 50 to 300 nm) (Kraft et al., 2014). Smaller PS can effectively boost cellular uptake efficiency, which is helpful to improve the therapeutic effect (Üner, 2016). In particular, using PDI to represent the PS distribution of the nanosuspension can effectively reflect the stability of the formulation. In general, the smaller the PDI value (< 0.3), the more uniform the particle size and the better the physical stability (Mura et al., 2021). In addition, it is generally recommended that the ZP value be around ± 30 mV, because strong static electricity can effectively hinder the aggregation of particles and maintain the steric stability of nanoparticles (Üner, 2016; Mura et al., 2021). Moreover, CU1-SLN has high EE% and DL%. The high EE% of the drug is considered to be a result of the lipophilicity of the drug and the high compatibility between the drug and lipid. In some reports, various SLNs have also been prepared and characterized. For example, Shrestha et al. (2021) prepared the SLN of phytosterol esters, the PS of the optimized formulation was (171 ± 9) nm and the EE% was (89 ± 5)%. The PS of the doxorubicin SLN prepared by Bayat et al. (2021) was 148.5 nm and the EE% was 86.1%. It was suggested that the CU1-SLN prepared in this study has superior performance at the formulation level. At last, in the *in vitro* release experiment, it may be because part of the drug was adsorbed on the surface of the nanoparticles, so it was able to enter the release medium faster in the first 8 h. However, the final result showed that CU1-SLN has a slow-release effect. Consequently, it is suggested that CU1-SLN can release the drug continuously for a long time, which may be a long-acting preparation.

To further evaluate the anti-tumor activity of CU1-SLN, the proliferation inhibitory effects of CU, CU1, and CU1-SLN on seven cell lines were determined. The results suggested that CU1 and CU1-SLN had a good inhibitory effect on six cancer cell lines, and these effects became more pronounced in a concentration- and time-dependent manner. After the structural modification, the inhibitory effect of CU on cancer cells was retained, and the effect of CU1 was similar to that of CU. Moreover, CU1-SLN greatly increased the activity of CU1 to inhibit tumor proliferation. The inhibitory effect of CU1-SLN on A549 at 48 h was already higher than that of the CU-loaded preparation designed by Feng et al. (2020) with the same excipients of HSPC and PVPk15 at 72 h (IC50: 45.23 μM vs. 53.56 μM). Furthermore, the toxicity of CU1 and CU1-SLN on normal liver cells L02 were significantly lower than that of CU, indicating that CU1 and its preparation improved the safety of CU, while also having a certain selective inhibitory effect on hepatoma cells.

A laser confocal microscope was used to observe the distribution of CU, CU1, and CU1-SLN in hepatoma SMMC-7721 cells and lung cancer A549 cells. The results showed that CU1 and CU1-SLN were mainly distributed in the nucleus of cancer cells like CU, suggesting that the drug may interact with the genetic material in the cell nucleus to achieve its anti-tumor effect. Meanwhile, CU1 and CU1-SLN were also distributed in the cytoplasm. It was speculated that CU1 and CU1-SLN may act on the mitochondria in the cytoplasm to exert anti-tumor effects. In apoptosis experiments, it was revealed that all three drugs could promote the apoptosis of these two cell types. Under the same concentration of drugs, the apoptotic rate of the CU1 group was higher than that of the CU group, which was consistent with the MTT experiment results. This finding suggests that structural modification improves the anti-tumor effect of CU and the selectivity of hepatoma and lung cancer cells. The apoptosis rate of CU1-SLN showed an absolute advantage in both cell lines, which may be related to the higher biological affinity of SLN.

## 5. Conclusion

This study combined structural modification with a nano-drug system. On the one hand, CU1 was successfully synthesized to protect the drug from rapid degradation, improve or preserve the inhibitory effect of the drug on cancer cells, while simultaneously reducin the toxicity to normal cells. In particular, CU1 showed higher safety. On the other hand, encapsulating CU1 in SLN with HSPC and PVPk15 as carriers increased the stability of the drug, and significantly improved the anti-tumor activity *in vitro*. However, the specific anti-tumor mechanism and the anti-tumor evaluation in animal models of CU1 and CU1-SLN needed to be further studied.
